# Referral Criteria from Community Clinics to Pediatric Emergency Departments

**DOI:** 10.1100/tsw.2008.38

**Published:** 2008-04-14

**Authors:** Jacob Urkin, Ilan Segal, Nurit Barak, Joseph Press

**Affiliations:** ^1^ Primary Care Unit, Faculty of Health Sciences, Ben-Gurion University of the Negev, Beer-Sheva, Israel; ^2^ Division of Pediatrics, Soroka University Medical Center, Beer-Sheva, Israel; ^3^ Clalit Health Services Child Health Center, Ofakim, Israel

**Keywords:** child health, pediatrics, emergency medicine, community medicine, health services management, referral

## Abstract

Referral of patients to a pediatric emergency department (PED) should be medically justified and the need for referral well communicated. The objectives of this paper were (1) to create a list of criteria for referral from the community to the PED, (2) to describe how community physicians categorize their need for referral, and (3) to determine agreement between the physician's referral letter and the selected criteria. We present a descriptive study of referrals to the PED of Soroka University Medical Center, Beer-Sheva, Israel, during February to April 2003. A list of 22 criteria for referral was created, using the Delphi method for reaching consensus. One or more criteria could be selected from this list for each referral, by the referring community physicians and, independently, based on the physicians' referral letters, by two consultants, and compared. There were 140 referrals included in the study. A total of 262 criteria for referral were selected by the referring community physicians. The criteria most frequently selected were: “Need for same-day consultation/laboratory/imaging result not available in the community” (32.1%), “Suspected life- or organ-threatening infection” (16.4%), and “Need for hospitalization” (15.7%). Rates of agreement regarding criteria for referral between the referring physicians and the two consultants, and a senior community pediatrician and a senior PED pediatrician, were 57.9 and 48.6%, respectively. We conclude that the standard referral letter does not convey in full the level of need for referral to the PED. A list of criteria for referral could augment efficient utilization of emergency department services and improve communication between community physicians and the PED.

## INTRODUCTION

Referral of a child from the community to the pediatric emergency department (PED) is the end result of some medical encounters. Such a referral involves issues of medical decision making as well as issues of health care policy and regulation of health care expenditure. By referring a child to the PED, the primary care physician is transferring some of the burden of diagnosis and treatment to the PED, while still retaining his position as the child's principal provider. A referral can be regarded as a request for help from the PED. Thus, the referring physician, as well as the child, becomes the PED’s client.

Before referring patients to the PED, primary care physicians are encouraged to adopt the role of gatekeepers and use resources within the community[1]. Inappropriate referrals increase the PED workload unnecessarily and delay treatment for urgent patients[2]. Tests and procedures done in the community are commonly repeated in the PED[3] and follow-up by the community physician after the PED visit is incomplete[4]. Thus, the use of PED for nonurgent medical problems is fiscally imprudent and medically undesirable[5,6]. It is, therefore, advisable to limit referrals to the PED to problems that necessitate emergency or hospital technologies or expertise.

The reasons for referral are usually communicated to the PED staff by a referral letter, which is often not explicit and open to misinterpretation[3]. Better presentation of the need for referral, as viewed by the referring physician, may help the PED staff to provide services that satisfy the patient as well as the referring physician.

Previous studies have evaluated the appropriateness of pediatric hospitalization[7,8]. However, different tools are needed to evaluate the need for referral to the PED. The aims of this pilot study were (1) to create a list of criteria for referral of children from the community to the PED; (2) to describe the referring community physician's need for referral, using the list of criteria; and (3) to determine agreement between the referring community physician's referral letter and the criteria s/he selected from the list of criteria.

## METHODS

### Study Design

This is a descriptive pilot study aiming to create and evaluate a tool for expressing the need for referral from the community to the PED.

### Study Setting and Population

The study was conducted in the Soroka University Medical Center, Beer-Sheva, Israel. Participants were community physicians whose patients arrived in the PED with a referral letter during clinic working hours. Primary community physicians who also serve in the PED were excluded from the study, as were physicians whose patients arrived after clinic working hours. Data collection was conducted over a 2-month period (mid-February to mid-April 2003).

### Study Protocol

A set of guidelines for pediatric admission to the hospital was previously developed, based on an international evaluation tool – "Pediatric Appropriateness Evaluation Protocol"[7,9]. Five senior pediatricians, serving in the PED, the hospital, and the community, participated in three rounds of a Delphi process for reaching consensus[10]. They used the above-mentioned guidelines as a reference for creating a list of 22 general criteria for referral of children to the PED. A form containing this list was used in the study.

When a patient arrived at the PED during clinic working hours with a referral letter, the referring community physician was contacted by telephone. The nature of the study was explained and the physician's participation was requested. It was emphasized that (a) individual patient clinical information is not recorded and (b) individual physician’s clinical decision making is not examined. The form containing the list of 22 criteria was forwarded by fax to the referring physician, who selected the criteria for this particular referral and returned the form by fax. No limitation was imposed regarding the number of criteria selected for each referral, thus, many forms indicated more than one criterion.

Two consultants, the director of the PED and a head of a community pediatric clinic, independently selected criteria for referral according to the information provided by the referral letters. Patient and referring physician's identifying information, as well as referring physician's selected criteria for referral, were concealed. The referring physician’s and the consultants' forms were compared. Agreement was defined as at least one criterion selected by both the referring physician and a consultant.

### Data Analysis

The frequency of selected criteria is presented as a percent of the total number of forms. The chi-square test was used for comparison of qualitative parameters, such as physicians of different backgrounds; $p$ value $< 0.05$ was considered statistically significant.

The study was approved by the research committee of the Joyce and Irving Goldman Medical School of Ben-Gurion University of the Negev.

## RESULTS

Following a Delphi process, a list of 22 general criteria that define need for referral of children to the PED was created. A form containing the list of criteria was prepared. Examples for each criterion were provided in brackets, e.g., "Suspected life- or organ-threatening infection (sepsis, meningitis, ocular herpes, cellulites)". An "Other" option was provided to allow physicians discretion in defining additional criteria. The form included instructions for users and a space for comments. The list of criteria appears in Table 1.

There are approximately 225 visits per week to the Soroka University Medical Center PED during community clinic working hours. A convenient sample of 181 forms, representing 10% of the visits, was sent by fax to referring community physicians who agreed to participate in the study; 146 forms were returned, six were rejected due to technical problems. Therefore, 140 forms (77.3%) were included in the study.

Fifty-two (37%) of the participating referrals were generated by board certified pediatricians, 16 (11%) by board certified family physicians, and 72 (52%) by general practitioners. The most common clinical conditions that required referral to the PED, according to the letters of referral, were systemic signs of concern (e.g., high fever, restlessness; 56.4%), and gastrointestinal and respiratory signs and symptoms (32.8 and 27.1%, respectively).

A total of 262 criteria were selected by the referring physicians. The criterion most frequently selected was "Need for same-day consultation/laboratory/imaging result not available in the community" (32.1%; Table 1). Board certified physicians indicated the criteria "Inability to maintain fluid balance or intake of essential medication or food" and "Foreign body that was not fully extracted" significantly more often than general practitioners ($p = 0.05$ and $p = 0.03$, respectively). The expression "Parental demand" did not constitute one of the criteria for referral, but was mentioned 12 times as a comment. General practitioners used this expression more often than board certified physicians ($p = 0.08$).

Based on the referral letter, in 77.2% of the cases, at least one of the consultants selected the same criteria for referral as the referring physician, signifying agreement (Fig. 1). In 57.9% of the cases, agreement was between the referring physician and the head of a community pediatric clinic, and in 48.6%, between the referring physician and the director of the PED. In 29.3% of the referrals, both consultants agreed with the referring physician. Although not statistically significant, consultants’ agreement was higher with board certified physicians than with general practitioners. It should be noted that, in 22.8% of the cases, neither of the consultants could find in the referral letter any criterion for referral.

**TABLE 1**
**Frequency of Criteria for Referring Children to the PED**

| Criterion | N* | % |
|---|---|---|
| Need for hospitalization | 22 | 15.7 |
| Definite case of emergency | 3 | 2.1 |
| Physical trauma that might jeopardize life, organ, or future function | 10 | 7.1 |
| Exposure to life-threatening substance | 1 | 0.7 |
| Bleeding, uncontrolled or significant | 0 | 0 |
| Need for emergency surgical intervention | 18 | 12.9 |
| Suspected life- or organ-threatening infection | 23 | 16.4 |
| Child abuse or neglect | 4 | 2.9 |
| Road accident | 0 | 0 |
| Significant deviation of vital signs | 5 | 3.6 |
| Suspected failure of vital organs | 4 | 2.9 |
| Inability to maintain fluid balance or intake of essential medication or food | 17 | 12.1 |
| Acute deterioration of sensory/motor function or consciousness | 5 | 3.6 |
| Fever that requires immediate workup | 17 | 12.1 |
| Unexplained intense pain or irritability | 13 | 9.3 |
| Acute, severe psychiatric condition | 4 | 2.9 |
| Unstable condition that requires close medical/nursing supervision | 13 | 9.3 |
| Significant variation in laboratory result that might indicate a threat to life or organ | 9 | 6.4 |
| Foreign body that was not fully extracted | 4 | 2.9 |
| Need for same-day consultation/lab/imaging result not available in the community | 45 | 32.1 |
| Urgent need for in-hospital technology | 9 | 6.4 |
| Unexpected deterioration | 15 | 10.7 |
| Other | 9 | 6.4 |
| Parental demand | 12 | 8.6 |

* Total number of forms = 140; total number of criteria selected = 262; more than one criterion per form was allowed.

## DISCUSSION

Studies in the U.S. demonstrated that up to three-fourths of emergency department visits are for nonurgent problems that might be more effectively handled in a primary care setting[1]. A study reported by Kini and Strait found that almost 75% of PED patients did not call their primary care physician prior to arrival in the PED[11]. Most studies focus on utilization of the PED as a walk-in clinic for nonurgent problems and the impact of changes in community primary care pediatric health services on PED utilization[6,12,13,14]. Factors that influence PED utilization practices were found to include ethnic background, economic and insurance status, source and availability of primary care[14]. Studies evaluating the effect of prior approval in managed care programs found that this is not an effective long-term strategy for reducing PED utilization[15,16,17].

Few studies concentrate on the justification of referral from the referring physician's point of view. A previous study conducted in the Soroka University Medical Center reveals that the most common reason for referral (79%) is a "Severe/life-threatening condition" as perceived by the referring physician[3]. The

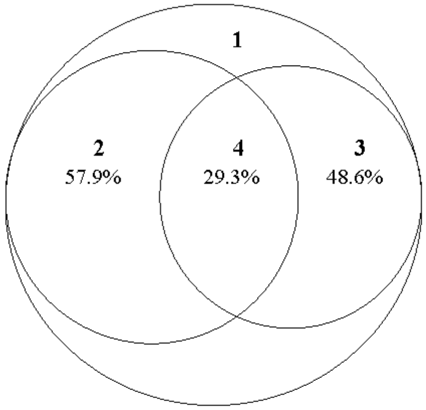

1 Total referrals (n = 140)
2 Agreement between the head of a community pediatric clinic and referring physicians
3 Agreement between the director of the pediatric emergency department and referring physicians
4 Agreement between both consultants and referring physicians

**FIGURE 1.** Agreement between referring physicians and consultants regarding criteria for urgency of referral.

second most common reason (10%) is the urgent need for same-day laboratory test or imaging procedure unavailable at the community setting.

Approximately 1,500 children visit the PEDs in Israel every day[18.] Due to Health Maintenance Organizations' copayment arrangements, most of these children are referred by their primary care physician with a referral letter[8].

The referral letter to the PED does not necessarily communicate the concerns of the referring physician. Failure to communicate the need for referral may cause inappropriate and inefficient utilization. In this study, a list of criteria for referral to the PED was developed. The study describes the criteria used by the referring physicians to justify referral, and the interpretation of the referral letters by two consultants.

While reviewing the results of this study, we made several observations. The criterion "Need for same-day consultation/laboratory/imaging result not available in the community" was the most frequently mentioned by the referring physicians (Table 1). As laboratory tests or imaging procedures are performed on almost all PED patients, this criterion is redundant. It would be preferable to denote the need for referral by specific clinical criteria. Therefore, this criterion should be eliminated from future versions of the list.

Any problem that justifies hospitalization is appropriate for referral to the PED[8]. Although the criterion "Need for hospitalization" seems too general, it relates to a specific list of criteria for pediatric admission from the PED to the hospital[9]. We suggest the use of this criterion together with an explanatory specific clinical criterion.

The category "road accident" was never selected in this sample since Soroka University Medical Center has a separate trauma department for accident victims.

Referral as a response to parental demand is often viewed as an example of PED misuse and as a sign of the physician's failure in his/her task as gatekeeper. However, this is not necessarily the case. Parents, as well as children, are the community physician's customers. Extreme parental anxiety over a child's health should be addressed and properly managed. A referral to PED is sometimes the best response.

The level of agreement between the criteria for referral selected by the referring physicians and the criteria selected by the two consultants, based on the referral letters, was 77.2%. However, in over 50% of the cases, the referral letters failed to convey the criteria for referral to the head of the PED. Agreement was higher (57.9%) with the community pediatrician, whose clinical thinking is probably closer to that of the referring physicians. The nonstructured referral letter is, therefore, not a very efficient method for communicating the need for referral from the community to the PED. Incorporating a list of criteria for referral in the referral letter could overcome this difficulty.

This study's limitations are the relative small sample size, gathered as a convenient sample of referrals and the reliance upon physicians' agreements to participate. Although it was specifically stated that the study would not evaluate individual parameters, we assume that some physicians declined to participate as it challenged their clinical decision making.

## CONCLUSIONS

We suggest that a list of criteria for referral should be incorporated in the referral letter to the PED. Health Maintenance Organizations and continuing medical education programs could use the information selected from this list to evaluate referral appropriateness and to plan educational interventions to community physicians. Use of the list of criteria for referral could improve communication between community primary care and PED physicians, and upgrade utilization of the PED.

## ACKNOWLEDGMENTS

The authors wish to thank Shirley Rosen, MSc, for statistical advice. The study received no financial support and no conflict of interest exists regarding this manuscript.
